# Specificity vs. Generalizability: Emergence of Especial Skills in Classical Archery

**DOI:** 10.3389/fpsyg.2016.01178

**Published:** 2016-08-05

**Authors:** Stanisław H. Czyż, Sarah J. Moss

**Affiliations:** ^1^Physical Activity, Sport and Recreation Research Focus Area, North-West UniversityPotchefstroom, South Africa; ^2^Department of Sport Didactics, University School of Physical Education in WrocławWrocław, Poland

**Keywords:** especial skill, motor learning, specificity of practice, variability of practice, conditions of practice, schema theory

## Abstract

There is evidence that the recall schema becomes more refined after constant practice. It is also believed that massive amounts of constant practice eventually leads to the emergence of especial skills, i.e., skills that have an advantage in performance over other actions from within the same class of actions. This advantage in performance was noticed when one-criterion practice, e.g., basketball free throws, was compared to non-practiced variations of the skill. However, there is no evidence whether multi-criterion massive amounts of practice would give an advantage to the trained variations of the skill over non-trained, i.e., whether such practice would eventually lead to the development of (multi)-especial skills. The purpose of this study was to determine whether massive amount of practice involving four criterion variations of the skill will give an advantage in performance to the criterions over the class of actions. In two experiments, we analyzed data from female (*n* = 8) and male classical archers (*n* = 10), who were required to shoot 30 shots from four accustomed distances, i.e., males at 30, 50, 70, and 90 m and females at 30, 50, 60, and 70 m. The shooting accuracy for the untrained distances (16 distances in men and 14 in women) was used to compile a regression line for distance over shooting accuracy. Regression determined (expected) values were then compared to the shooting accuracy of the trained distances. Data revealed no significant differences between real and expected results at trained distances, except for the 70 m shooting distance in men. The *F*-test for lack of fit showed that the regression computed for trained and non-trained shooting distances was linear. It can be concluded that especial skills emerge only after very specific practice, i.e., constant practice limited to only one variation of the skill.

## Introduction

The increased interest in mechanisms underlying the emergence of especial skills have resulted in a number of publication over the last decade (Keetch et al., 2005, 2008; Breslin et al., 2010; Czyż et al., 2013, 2015; Stöckel and Breslin, 2013). The especial skill is considered to have an advantage in performance over a class of actions executed by the same Generalized Motor Program (Keetch et al., 2005) however the mechanisms underpinning such an advantage is unclear. A few hypotheses on the underpinning mechanisms that result in this advantage have been proposed. These hypotheses include: learned-parameters hypothesis, visual-context dependency, especial Generalized Motor Program hypothesis (see Breslin et al., 2012b for review) or the mediating role of self-efficacy (Simons et al., 2009). All of these hypotheses have been tested in recent publications (see Breslin et al., 2012b for review; Simons et al., 2009; Czyż et al., 2015) providing contradictory findings.

Originally, Keetch et al. (2005) linked the presence of especial skills to the massive amount of practice. Based on a suggestion by Keetch et al. (2005) in follow-up studies it was assumed that a massive amount of practice is a crucial factor leading to the emergence of especial skills. This notion resulted in recruitment of participants with a high number of accumulated training hours (e.g., Fay et al., 2013) and/or several years of practice (e.g., Keetch et al., 2005; Simons et al., 2009).

On the contrary, Breslin et al. (2012a) suggested that the effect of especial skills is due to the manner of motor practice rather than (massive) amounts of practice itself. In their study, they evoked especial skills after 300 trials of constant practice. Also Czyż et al. (2013) suggested that especial skills may be present at early stage of learning and massive amount of practice is not a crucial factor for its emergence. Czyż et al. (2013) found that specificity of practice may result in especial skill at the very early stage of learning, however, a different model than the one proposed by Keetch et al. (2005) has to be used to detect them. The proposed model by Czyż et al. (2013) is based on an assumption that specificity and generalizability are two extremes of the same motor learning (see also Poggio and Bizzi, 2004). The assumption is that both processes take place at the same time, however, one of them is stronger depending on how much constant practice learners received. Czyż et al. (2013) were able to emulate the balance between these two common processes. As a result of this emulation, the model was capable of detecting especial skills at the very early stage of learning. Alike Breslin et al. (2012a); Czyż et al. (2013) did not find a correlation between amount of practice and especial skills.

Based on previous research on variability of practice (Van Rossum, 1990) and on Schmidt’s (1975, 2003). Schema theory, it can be assumed that variable practice favors generalization whereas constant practice, like in studies about especial skills (Breslin et al., 2012b), favors the specificity process. It is assumed that variability of practice should result in better learning, i.e., better retention (Schmidt and Lee, 2011) and in more accurate and stable performance (Lee et al., 1985; Breslin et al., 2012a). These assumptions are in line with Schmidt's (1975) schema theory and were confirmed in several studies (see Schmidt and Lee, 2011 for the review). Variable practice that involves practicing several variations of the movement leads to the more flexible and stronger movement representation (Breslin et al., 2012a). As a result, variable practice prepares better for novel situations, i.e., for transfer (Shea and Kohl, 1990, 1991). On the other hand, as it was shown in one of the classical experiments on variability of practice, the experiment of Shea and Kohl (1991), the criterion tasks is better performed when it is followed by constant practice involving criterion task compared to the same amount of criterion and variable practice.

In all of the studies that tested the benefits of constant vs. variable practice only the effect of one criterion task practice compared to one criterion + variable (e.g., Shea and Kohl, 1990, 1991; Breslin et al., 2012a) or variable practice (e.g., Shoenfelt et al., 2002) was examined. There are limited studies testing the multiple criterion practice indicating a need for investigations to determine if multi-criterion practice leads to development of (multi) especial skills. Findings from such an investigation may help us to estimate how specific should practice be to give an advantage of specificity over generalizability.

The benefits of constant practice, as pointed out by Keetch et al. (2005) and Breslin et al. (2012a), is that after constant practice the recall schema becomes more refined. This is probably why especial skills have an advantage over the actions from within the same class of actions. As it was shown in the model by Czyż et al. (2013), the specificity process was stronger in especial skills than generalization. However, generalization was also observed. The Breslin et al. (2012a) results also yielded that there is some generality within class of actions even after shooting at free throw distance in basketball, i.e., after constant practice. Would the specificity of practice be noticed if the acquisition of the skill involved more than one criterion variation? Answering this question could have a strong implication on practice as well as on theory of motor learning. The answer could show the relation between both processes in motor learning: specificity and generalizability.

In all of the recent studies focusing on especial skills, only skills that were massively practiced in one criterion variation were tested. It was either the basketball free throw (e.g., Breslin et al., 2012a; Czyż et al., 2013) or baseball throw (Keetch et al., 2005; Simons et al., 2009).

In this study we would like to determine whether massive amounts of practice involving more than one criterion variation of the skill will also give an advantage to the criterion over the class of actions.

The authors could not find studies which investigated especial skills, on the multi-criterion variations of the skill. We therefore undertook a study to investigate how specific practice should be for the especial skill(s) to emerge. We conducted our study on classical male and female archers. According to the World Archery Federation Rule Book 2 (2015), men and women compete on four official distances. Therefore, they train four variations of the skill. For men these distances are: 30, 50, 70, and 90 m whereas for women 30, 50, 60, and 70 m. As a result we could test four criterion variations of the skill against non-trained variations. We recruited experienced participants, so we could also test if the relation between amount of practice and especial skill is present.

## Materials and Methods

Two experiments were conducted with the shooting scores as the dependent variable and distances as independent variables. The purposive sampling method was applied.

Permission for the subjects to participate in both experiments described in this manuscript was specifically approved and granted by the Committee for Ethics of the University School of Physical Education in Wroclaw, Poland. Research was conducted according to the declaration of Helsinki and adhered to The Ethical Principles and Guidelines for the Protection of Human Subjects of Research (commonly called the Belmont Report) promulgated in 1979. All participants took part in the study on a voluntary basis and could discontinue their participation at any time without any consequences.

### Experiment 1

#### Method

##### Participants

Ten males participated in the study. Four participants were from Poland, two from Peru, and one each from Argentina, Colombia, and Mauritius (**Table 1**). Since archery is a niche sport, it was not possible to recruit a sufficient sample of experienced archers from one country. We decided to invite archers from all over the world using personal emails as well as social platforms dedicated to archery. Of the 13 experienced archers that agreed to take part in our study only 10 participants completed the study. The reason for resigning from the study was indicated as being asked to perform archery shots from distances that they never shoot on official competitions and that completing the requested task would interfere with their training schedule.

**Table 1:** Descriptive statistic (mean and SD) of male participants from Experiment 1.

Archer	Country	Age (years)	Estimated accumulated training hours	Total score
1	Mauritius	24	6552	918
2	Colombia	20	3900	1135
3	Morocco	27	4992	982
4	Peru	17	832	889
5	Peru	19	832	783
6	Argentina	41	3900	1020
7	Poland	20	7488	1034
8	Poland	20	4992	1017
9	Poland	18	2496	770
10	Poland	21	7280	936
	Mean	22.70	4326.40	948.40
	*SD*	6.69	2295.53	113.97

Mean age of the participants was 22.7 years (*SD* = 6.69), with an average of 5.2 (*SD* = 2.71) years of training. The estimated mean accumulated practice hours of archery was calculated as number of years training archery, multiplied by 52 weeks, multiplied by self-reported hours of training per week. This calculation indicated an estimated mean of 4326.4 h (*SD* = 2295.5). This assessment method was previously used by Ford and Williams (2008) and Fay et al. (2013).

One of the participant was Academic World Vice-Champion, one was European championship medalist, five were national champions, two were national championship silver medalists, and one was regional champion.

##### Task and procedure

Participants performed 30 shots from 20 different distances, i.e., 600 shoots in total. According to World Archery Federation [*Fédération Internationale de Tir à l’Arc* (FITA) World Cup men shooting includes abovementioned four distances] male classical archers shoot at four competition distances, i.e., 30, 50, 70, and 90 m. Therefore, participants were asked to shoot from these four accustomed (trained) distances as well as from non-trained distances at 22, 26, 34, 38, 42, 46, 54, 58, 62, 66, 74, 78, 82, 86, 94, 98 m respectively. As a result, we had 20 shooting distances 4 m apart. According to World Archery Federation (WA) men shoot on the 80 cm diameter face at 30 and 50 m distances and on a 122 cm diameter face at 70 and 90 m. Therefore, we asked participants to shoot on an 80 cm diameter face at distances between 22 and 58 m and on a 122 cm diameter face at distances between 62 and 98 m.

We conducted a two-subject pilot study (not included in the further data analysis) to assure that the number of shots that can be taken on three consecutive days without any substantial technical or physical problems. We also wanted to confirm that 600 additional shots per 3–4 days will not be more physically demanding than ordinary training. The following shooting procedure was followed according to suggestions made by experienced archery trainers. Participants were requested to perform all shots on three-four consecutive training sessions in a blocked order. This procedure reduced the time it takes to adjust the sight on the bow and set the face for each of the particular distances.

Three Master students collected data in Poland. Participants from other countries were provided with the questionnaires and the description of the procedure via email. A pre-modified Excel sheet to record the shots’ scores consisting of a table and shooting instruction were included in the sent email. The questionnaire was a self-reported English questionnaire collecting information on the age, country of origin, details about training habits (hours per week, years of training, preferable shooting distances, the worst shooting distances) and achievements (participation in Olympics, world, continental, or national competitions) of the participants. Results from the shots were sent to us electronically.

##### Data analysis

**FIGURE 1:**
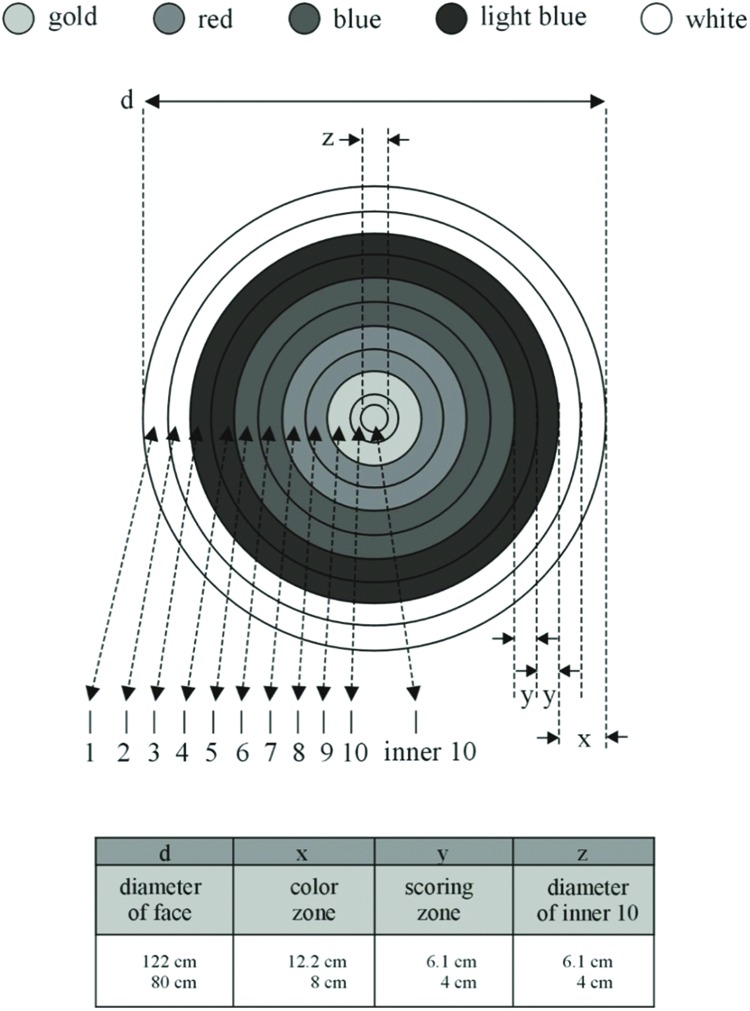
**The 1–10 scoring zone target face (according to World Archery Federation Rule Book 3, 2015)**.

The outcome scores at each distance were calculated according to the World Archery Rules Book^1^ (**Figure 1**). Participants were asked to perform all shots on the range they train according to FITA rules and their ordinary training routine. The final scores as the sum of points gained on 30 shots at a distance were included in the statistical analysis. Participants used arrows and bows according to WAR recognized by and a core sport of the International Olympic Committee.

The especial skill effect was tested using Keetch et al. (2005) method, i.e., *t*-test were used to compare expected scores to the real scores noticed in the experiment at same distances. The expected scores were calculated based on regression lines fitted to the data for each participant. We used the *F*-test for lack of fit to determine whether our data can be approximated by a linear regression.

#### Results and Analysis

**FIGURE 2:**
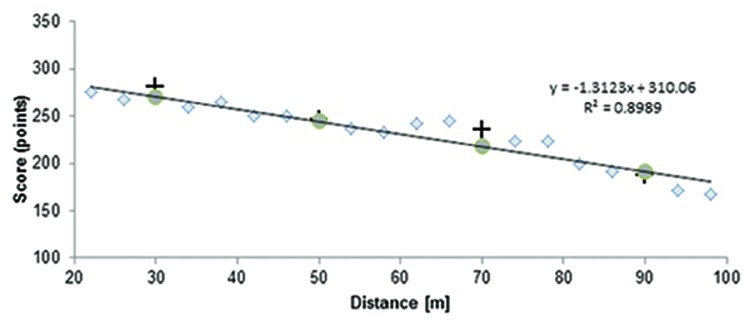
**The linear regression computed for non-trained distances**. The gray diamonds reflect the average scores at non-trained distances, crosses reflect average scores at trained distances, and gray circles predicted scores at distances of 30, 50, 70, and 90 m.

We calculated a shot score across each block of 30 shots at each distance for each participant and an average shot score for all participants. We used a similar method of detecting especial skills as was originally used by Keetch et al. (2005). A linear regression was computed for scores at non-trained distances (**Figure 2**), i.e., for all distances but 30, 50, 70, and 90 m.

We calculated an expected shot score at 30, 50, 70, and 90 m based on a linear regression equation for each participant (see Experiment 1 in Keetch et al., 2005; Czyż et al., 2013; Fay et al., 2013). The average expected score was compared to the real score at the distances of 30, 50, 70, and 90 m in one-tailed paired samples *t*-test.

There were no statistically significant differences between real and expected scores at 50 m [one tailed *t*(9) = 0.24; *p* = 0.47] and at 90 m [one-tailed *t*(9) = -0.83; *p* = 0.21]. We found significant differences between real and expected scores at 30 m [*t*(9) = 1.87; *p* = 0.047] and 70 m [*t*(9) = 4.50; *p* = 0.001]. Due to the fact that we were testing hypotheses using multiple comparison, we applied the Bonferroni correction, i.e., we tested hypotheses at the level of *p* = 0.05 divided by the number of comparisons, i.e., four. As a result, we set our significance level at *p* = 0.012 that equaled *p* = 0.05 in a single comparison. The only significant difference after having applied the Bonferroni correction was the difference between real and expected values at 70 m. The effect size was also medium (Cohen’s *d* = 0.50).

These results do not prove that specific practice limited to four trained *parameters* – *outcome* relations, leads to the emergence of especial skills. Originally, Keetch et al. (2005) argued that a massive amount of practice leads to the emergence of an especial skill. However, Czyż et al. (2013) argued that not massive amount of practice but the general proficiency is correlated with the especial skill effect.

**FIGURE 3:**
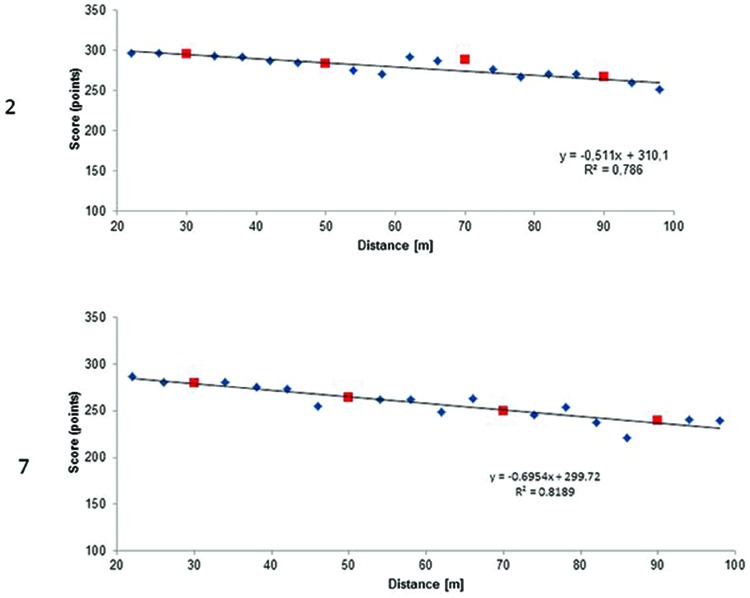
**Regression lines for participants numbers 2 and 7 (see Table 1) who achieved highest scores (1135 and 1034 h respectively).** The squares represent scores at 30, 50, 70, and 90 m and small diamonds the remaining distances.

**FIGURE 4:**
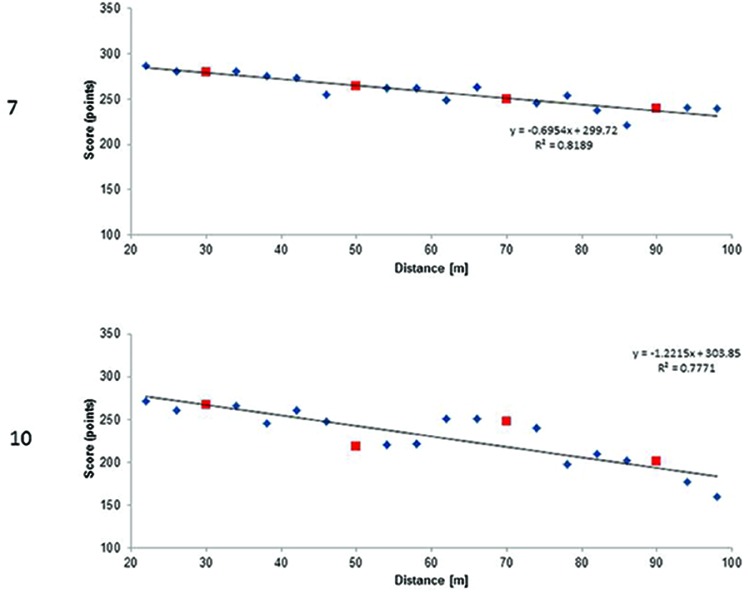
**Regression lines for participants numbers 7 and 10 (see Table 1) who reported the highest estimated accumulated training hours (7488 and 7280 h respectively).** Small diamonds represent scores at all distances but 30, 50, 70, and 90 m. These latter ones are represented by squares.

Given that our group of participants differed in terms of experience and achievements, we decided to analyze the data of both the players that obtained the best total scores (achieved in the test) and the highest number of accumulated training hours. Czyż et al. (2013) reported strong correlation between general proficiency and especial skill. The general proficiency in archers can be represented as a total shooting score calculated as a sum of scores achieved at 30, 50, 70, and 90 m distances. In **Figure 3** we present the regression lines computed for non-trained shooting distances scores for two archers that achieved the best total scores. In **Figure 4** we present regression lines for two archers who had the highest number of estimated accumulated training hours. These latter cases test the Keetch's et al. (2005) hypothesis about the relation between massive amount of practice and the presence of especial skill.

The first impression while analyzing functions in **Figure 3** is that the regression in participant 2 was almost linear, including scores obtained at distances 30, 50, 70, and 90 m. Real scores obtained during the tests and expected values computed based on the participant’s linear regression analyses are presented in **Table 2**. The expected scores for the trained shooting distance, i.e., 30, 50, 70, and 90 m were calculated based on the participant’s linear regression equation. Regarding participant 2, the differences between expected and real scores were very small, and at the 50 m distance the expected score was higher than the real score. On the other hand, in participant 7, the differences between real and expected scores at these distances were very small, and at 50 and 70 m the expected scores were higher than the real scores. Especial skill is therefore not observed at these distances. Since especial skill has a unique place within a class of movement, it is therefore difficult to claim, that 50 or 70 m scores could reflect especial skill. The score at 90 m in participant 7 is rejecting the hypothesis.

**Table 2:** Real and expected scores achieved at distances of 30, 50, 70, and 90 m for participants numbers 2 and 7 with the best scores reported in the questionnaire.

Distance (m)	Participant 2		Participant 7	
	Real	Expected	Real	Expected
30	296	294.8	280	278.86
50	284	284.6	264	264.95
70	288	274.3	250	251.04
90	267	264.1	240	237.13

Analogically, we computed regression lines for participants with the highest number of accumulated training hours (**Figure 4**). Whereas the regression in participant 7 was almost linear, the regression in participant 10 was non-linear.

Again, we calculated expected scores for the trained shooting distance, i.e., 30, 50, 70, and 90 m based on the participant’s linear regression equation (**Table 3**). In participant 10, the expected score at 50 m was higher than the real score. However, the real score at 70 m was higher than the expected score and with the points representing scores from neighboring distances formed a kind of generalization gradient. This finding would be in line with Czyż et al. (2013; see also Fay et al., 2013) who confirmed Rosenbaum’s idea (Keetch et al., 2005) that an especial skill may create a generalization gradient during its development.

**Table 3:** Real and expected scores achieved at distances of 30, 50, 70, and 90 m for participants numbers 7 and 10 with the highest number of accumulated training hours.

Distance (m)	Participant 7		Participant 10	
	Real	Expected	Real	Expected
30	280	278.86	268	267.21
50	264	264.95	219	242.78
70	250	251.04	248	218.35
90	240	237.13	201	193.92

However, it is always more difficult to prove that something does not exist than *vice versa*. Therefore, we decided to perform the *F*-test for lack of fit. This test determines whether a specific type of regression function adequately fits the data (Kutner et al., 2006). We assumed that if the specificity is stronger than generalizability in our participants, the regression computed for the shot efficiency scores at all distances, will not be linear. Specificity of practice should curve the function at the shooting distances that were massively trained. As a result, this test could help us to specifically determine whether there is non-linearity in our data that may be associated with specificity of practice.

The null hypothesis (H_0_) of this test assumes that the regression function is linear, whereas the alternative hypothesis (*H*_α_) assumes that the regression is non-linear.

The *H*_0_ can be concluded when *F^∗^*-value is smaller or equal to *F*(1-*α*; c-2, n-c)-value, where *c* is the number of levels (in our case 20 different shooting distances), and *n* is the number of observations (200 in our experiment). The *H*α can be concluded when the *F*^∗^-value is higher than the *F*(1-*α*; c-2, n-c)-value.

To calculate the *F*^∗^, we computed regression lines across the scores for all distances. The regression equation obtained was:

(1) y=-1.3397⁢x+312.86.⁢                          (1)

The regression accounted for 90% of data variability (*R*^2^ = 0.90).

The *F*^∗^-value for our lack of fit test was *F*^∗^(18,180) = 1.76.

If the level of significance is to be α = 0.01 then *F*(1-α; c-2, n-c) is in our case equal to *F*(0.99;18,180) = 2.04.

The *F*^∗^ is smaller than *F* therefore we can conclude *H*_0_, i.e., the regression function is linear. The *p*-value for our test is 0.03.

This test eventually confirmed our first visual impression (see **Figure 2**) that our data can be approximated by linear function.

Based on the *F*-test for lack of fit result, we conclude that there was no especial skill effect in our participants with the selected experimental setup. The conclusion should be interpreted against the limitations of the study design which required self-reported results on the shots performed that require honesty and sincerity from the participants. Although self-reported methods are widely used in scientific research, a well- controlled study was needed to verify the findings of this experiment. Therefore, experiment 2 was conducted.

### Experiment 2

The following experiment was conducted to verify our findings reported in Experiment 1. In this experiment new participants were recruited and only female archers were included.

#### Method

##### Participants

The participants consisted of eight Polish female archers (**Table 4**). The mean age of the participants were 24.6 years (*SD* = 3.5), with an average 10.4 (*SD* = 3.1) years of training. The estimated mean accumulated practice hours of archery was estimated as the number of years of training archery multiplied by 52 weeks, multiplied by self-reported hours of training per week. The estimated mean accumulated practice hours were 7943.0 h (*SD*: 5906.7; see **Table 4**). Two of the participants were Olympic athletes, one bronze World Championship medalist and all of them but one, Polish champions (individually, mixt, or team). All of the participants were Polish national team members. The least skilled participant was fourth in Polish Championship and Polish Cup.

**Table 4:** Descriptive statistics of female participants in Experiment 2.

Archer	Age (years)	Years of training	Training hours per week	Estimated accumulated training hours	Total score
1	23	12	25	15600	1111
2	25	8	7	2912	1141
3	25	8	7	2912	1111
4	20	9	10	4680	986
5	27	8	7	2912	962
6	24	13	10	6760	1032
7	21	8	20	8320	970
8	32	17	22	19448	1109
Mean	24.63	10.38	13.50	7943.00	1054.4
*SD*	3.50	3.12	7.05	5906.74	67.50

*SD, standard deviation.*

##### Task and Procedure

Three Master students assisted with the data collection.

We followed the same procedure as in Experiment 1, however, women shoot from four distances; namely 30, 50, 60, and 70 m respectively (official distances according to WA). Therefore, participants were asked to perform additional shots from non-trained distances at 22, 26, 34, 38, 42, 46, 52.5, 55, 57.5, 62.5, 65, 67.5, 72.5, 75 m. The shooting distances were located 4 m from each other for distances closer and equal to 50 m. Shooting at distances further than 50 m were increased by 2.5 m up to 75 m. The rationale for these differences were to ensure equal distances between the subsequent distances of shooting.

Participants performed a total of 540 shoots (30 shots from 18 different distances).

According to WA rules women shoot on an 80 cm diameter face at 30 and 50 m distances, whereas on 60 and 70 m distances are shot at on a 122 cm diameter face. Participants were therefore asked to shoot on 80 cm face at distances between 22 and 55 m and on 122 cm face at distances between 57.5 and 75 m.

##### Data analysis

The outcome scores at each distance were calculated according to the WA Rule Book^2^ (**Figure 1**). Similar as in Experiment 1 participants were asked to perform all shots on the range they train according to FITA rules and their ordinary training routine. The final scores as the sum of points gained on 30 shots were included in the statistical analyses. Participants used arrows and bows according to WA standards and rules.

The especial skill effect was tested using Keetch et al. (2005) method, i.e., *t*-test were used to compare expected scores to the real scores recorded in the experiment at the corresponding distances. The expected scores were calculated based on regression lines fitted to the data of each participant.

#### Results and Analysis

**FIGURE 5:**
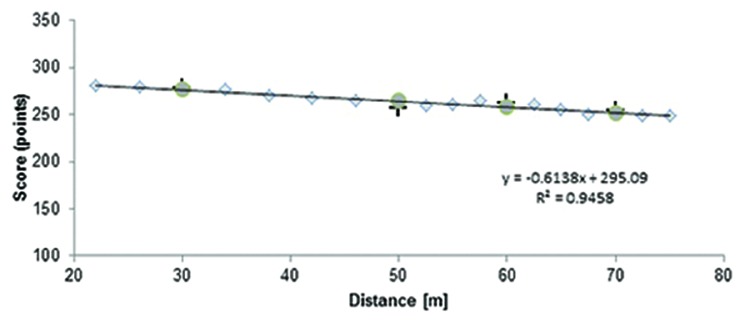
**The linear regression computed for non-trained distances for female archers in Experiment 2.** The gray diamonds reflect the average scores at non-trained distances, crosses reflect average scores at trained distances, and gray dots predicted scores at distances of 30, 50, 60, and 70 m.

Similar to Experiment 1 a shooting score across each block of 30 shots at each distance for each participant was calculated and an average shooting score for all participants calculated (**Table 1**). A linear regression was computed to approximate all scores at non-trained distances (**Figure 5**), i.e., for all distances but 30, 50, 60, and 70 m.

We calculated an expected shot score at 30, 50, 60, and 70 m based on the linear regression equation obtained for each participant. The average expected score was compared to the real score at the distances of 30, 50, 60, and 70 m in one-tailed paired samples *t*-test.

There were no significant differences between real and expected scores at 30, 60, and 70 m [one-tailed *t*-test: *t*(7) = 0.51, *p* = 0.31; *t*(7) = 1.51, *p* = 0.08; *t*(7) = 1.32, *p* = 0.11; respectively].

There was a significant difference at 50 m distance, *t*(7) = -2.97, *p* = 0.01. However, after having applied Bonferroni correction (see Experiment 1) no significant difference between real and expected scores was noticed.

In further analyses the individual regression lines for participants that achieved the highest total score as well as those who accumulated the highest number of training hours were performed (**Table 5**).

**Table 5:** Real and predicted scores achieved at distances of 30, 50, 60, and 70 m for participants number 2, 3, 4, and 10.

Distance (m)	Participant 1		Participant 2		Participant 3		Participant 8	
	Real	Expected	Real	Expected	Real	Expected	Real	Expected
30	293	294.04	295	291.71	289	286.28	292	285.92
50	272	282.57	285	283.94	270	278.10	272	274.43
60	273	276.84	284	280.05	275	274.00	278	268.68
70	273	271.10	277	276.16	277	269.91	267	262.93

**FIGURE 6:**
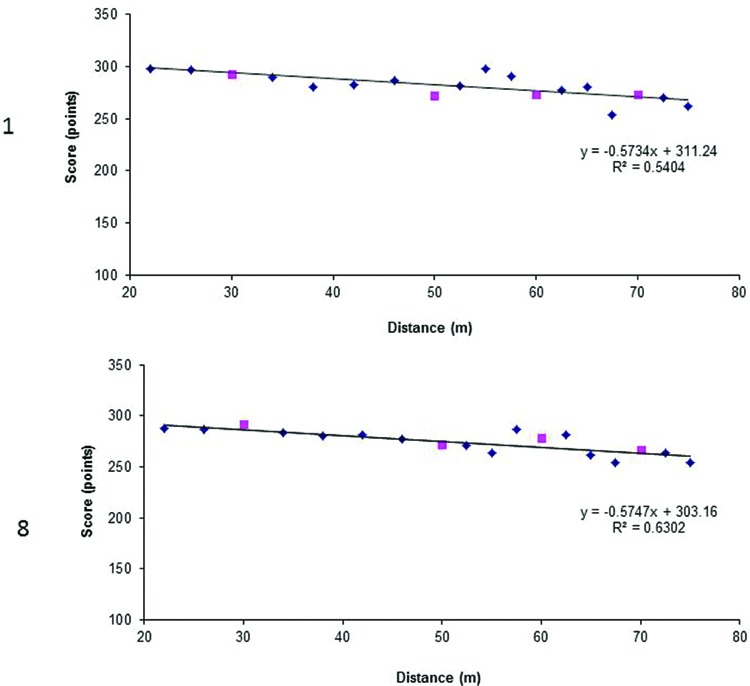
**Regression lines for participants 1 and 8 whom accumulated the highest number of training hours according to the self-reported questionnaire.** Diamonds reflect scores at non-trained distances, whereas squares at trained distances, i.e., 30, 50, 60, and 70 m.

**FIGURE 7:**
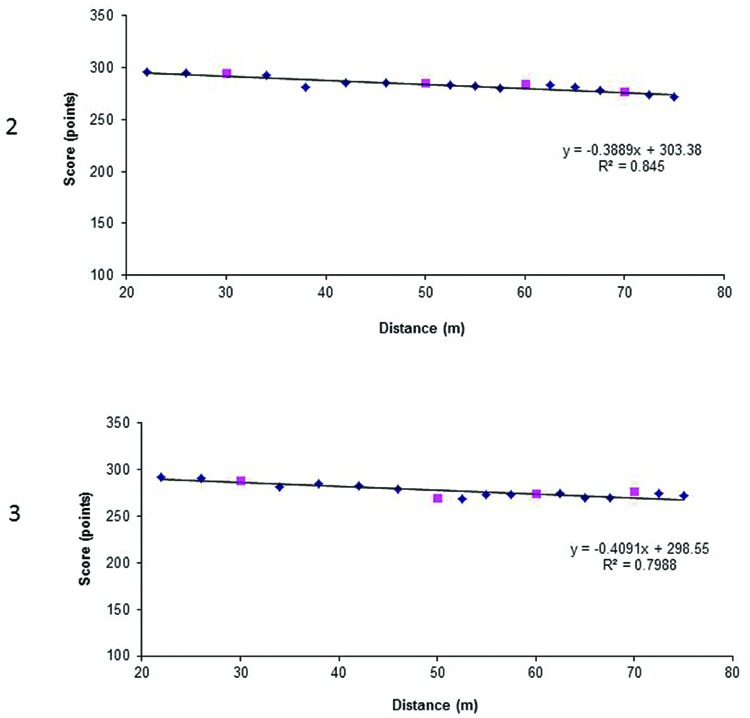
**Regression lines for participants 2 and 3 whom achieved the highest total scores, calculated as the sum of scores achieved at 30, 50, 60, and 70 m.** Diamonds reflect scores at non-trained distances, whereas squares at trained distances, i.e., 30, 50, 60, and 70 m.

In **Figure 6** we see regression lines for participants 1 and 8, who accumulated 15600 and 19448 h, respectively with both regressions almost linear. At some of the distances the expected scores were even higher than the real scores. On the other hand, in **Figure 7**, we presented the regressions lines for archers who achieved the highest total scores. Participant 2 achieved 1141 points, and participants 1 and 3 a total of 1111 points. Since the lines for participant 1 is already presented in **Figure 6**, only lines for participants 2 and 3 are presented in **Figure 7**.

The linear regression equation we computed for all participants accounted for 94% of data variability.

Finally, as in Experiment 1, we decided to perform the *F*-test for lack of fit to determine whether our data can be approximated by linear regression.

We computed the regression line for scores at all distances:

(2) y=-0.6018⁢x+294.55.⁢                          (2)

The coefficient of determination was even higher than in Experiment 1 and accounted for 91% of data variability (*R*^2^ = 0.91).

The *F*^∗^-value for our lack of fit test was *F*^∗^(16,144) = 0.22.

If the level of significance is to be α = 0.01 then *F*(1-α; c-2, n-c) is in our case equal to *F*(0.99;16,144) = 2.13.

The *F*^∗^ is smaller than *F* therefore we can conclude *H*_0_, i.e., the regression function is linear (*p* = 0.99; see **Figure 5**).

## Discussion

We examined whether specific multi-criterion practice that included practice of four skill variations gives an advantage to the practiced variations over non-practiced, i.e., leads to the development of what could be defined as especial skills. In two experiments we showed that especial skill is not present after massive specific practice. We did not notice any advantages of massive training when comparing the scores achieved at trained and non-trained shooting distances in archers except for shooting at 70 m distance in men (Experiment 1). However, in both experiments the mean scores across all shooting distances, including non-trained and trained distances could be approximated by linear regressions. It could be recapitulated that in our participants the generalizability was stronger than the specificity effect. In previous studies, especial skills were evoked following the practice of one skill variation as presented in either shooting at free-throw distance in basketball or in baseball (Keetch et al., 2005; Simons et al., 2009). Our data with archers show that if the practice is more variable, however, still very specific, i.e., includes four criterion variations only, the especial skill effect dissipated.

Since we have found significant difference between expected and real scores at 70 m distance in men, it could be claimed that there was “especial skill” effect. However, we have assumed that our participants massively trained on all four distances, i.e., 30, 50, 70, and 90 m. In classical archery, archers compete on four distances. It refers to all competitions, including world championships. However, the one competition that is organized differently is the Olympic Games. During Olympics, archers shoot at only one distance, i.e., 70 m. We think that this substantial difference at the distance of 70 m could be explained in the view of Olympic Games competitions. It could be speculated, that our participants received more practice at this distance. As a result, the difference between expected and real scores at 70 m was statistically significant. However, it is only a speculation and it needs to be confirmed. Hence, the question why the especial skill emerged only at the 70 m distance remains unclear.

Our finding is in line with previous studies on variability of practice. For example in the Shea and Kohl (1991) study, the best results were achieved by participants who in the acquisition phase practiced criterion task compared to the participants that received the same amount of practice in total but it included practice of criterion task as well as other tasks. As Schmidt and Lee (2011, p. 367) point out “*(...) practice task that were similar to the criterion task actually facilitated its retention.*” This statement was confirmed in the previous studies on especial skill in which basketball and baseball throwing at one distance was tested. Practice at tasks that included one variation of the skill and was the criterion (shooting at one distance) facilitated the memory representations, i.e., refined the recall schema within a group of skills (Breslin et al., 2012a). However, in our study, massive amount of practice that involved four variations of the skill, all of them were criterion tasks, did not lead to the emergence of especial skills. In other words, massive amount of practice did not give an advantage to the trained (criterion) tasks over non-trained tasks. It seems that more variable practice has not favored trained tasks but rather facilitates development and retention of the whole schemata, including non-criterion (non-trained) variations.

The schema theory by Schmidt (1975, 2003) suggests the same finding: no single action within a class of action can be more refined than others from the same class (Breslin et al., 2012a). So maybe, the specificity of practice effect can be noticed exclusively when extremely specific practice takes place and one variation of the skill (criterion task) is practiced (Keetch et al., 2005, 2008; Simons et al., 2009).

Our results indicate that the manner of the practice, more specific or more variable will actually favor the generalization or the specificity process. Based on our results, it can be suggested that specific massive amount of practice including four skill variations will favor generalizability over specificity.

Our findings should be interpreted against certain limitations. Firstly, since a limited number of participants were available in one country, data collection for Experiment 1 was based on self-reports. Although, this technique of collecting data is widely used, e.g., in retrospective research on expertise in sport, caution should be exercised. Secondly, test order may affect our results. In this study we tested our participants in a blocked schedule. In a randomly ordered test we could expect a dramatical drop-off in performance, but only if the randomly ordered retention tests were following the blocked practice. In contrast, the blocked-ordered retention trials would result in slightly lower scores, if the acquisition phase was ordered in blocked manner (Shea and Morgan, 1979; Lee, 2012). We do not know how the acquisition phase was scheduled; therefore, a hypothetical influence of contextual interferences is unknown.

## Conclusion

We showed that no single action within a class of actions can be more refined than others from the same class after following massive amount of four criterion task practice of classical archers. There may be some outperformed tasks in the four-criterion practice schedule; however the nature of this advantage is unknown and may be attributed to the personal schedule differences. We showed that when four criterion tasks are practiced the generalizability mechanisms are stronger than the specificity mechanisms. Our results also suggest that practicing four variations of the task, even when all of them are criterion tasks, is not specific enough for especial skill to emerge. This finding supports the generalizability view of motor learning that refers to Schmidt’s (1975, 1980, 2003) schema theory.

## Author Contributions

SC has contributed to the conception and design of the work, data collection, performed statistical analyses, and drafted the manuscript. SM contributed to the work by supporting the conception, designing and revising the work critically. All authors read and approved the final manuscript.

## Conflict of Interest Statement

The authors declare that the research was conducted in the absence of any commercial or financial relationships that could be construed as a potential conflict of interest.

## Footnotes

1 www.worldarchery.org

2 www.worldarchery.org
